# The brief psychotherapeutic intervention “relaxation, mental images and spirituality”: a systematic review

**DOI:** 10.1590/1516-3180.2019.030202102019

**Published:** 2020-04-03

**Authors:** Carlene Souza Silva Manzini, Vanessa Almeida Maia Damasceno, Ana Catarina Araújo Elias, Fabiana de Souza Orlandi

**Affiliations:** I MSc. Nurse and Doctoral Student, Department of Nursing, Universidade Federal de São Carlos (UFSCar), São Carlos (SP), Brazil.; II MSc. Physiotherapist and Doctoral Student, Department of Nursing, Universidade Federal de São Carlos (UFSCar), São Carlos (SP), Brazil.; III PsyD. Psychologist, Department of Psychology, and Full Professor of the Psychology Course, Universidade Paulista (UNIP), Campinas (SP), Brazil.; IV DNP. Nurse and Adjunct Professor IV, Department of Gerontology, Universidade Federal de São Carlos (UFSCar); and Permanent Professor of the Postgraduate Nursing Program at UFSCar, Sao Carlos (SP), Brazil.

**Keywords:** Complementary therapies, Psychosomatic medicine, Palliative care, Complementary medicine, Alternative therapies, Guided images, Directed imagination, Visualization

## Abstract

**BACKGROUND:**

The brief psychotherapeutic intervention “relaxation, mental images and spirituality” (relaxamento, imagens mentais e espiritualidade, RIME) is a form of complementary and alternative health-related therapy. It is a pioneer in the matter of relating the elements of spirituality to relaxation and to visualization of mental images.

**OBJECTIVE:**

To ascertain the history, use and benefits of RIME that have been reported in the scientific literature, within different health/disease contexts. The questions that guided this study were: In what contexts has the brief RIME psychotherapeutic intervention been used? What were its benefits?

**DESIGN AND SETTING:**

Systematic review, conducted in accordance with the Preferred Reporting Items for Systematic Reviews and Meta-Analyses (PRISMA) methodology, in a public university.

**METHODS:**

The BVSPsi, CINAHL, MEDLINE, SciELO, SCOPUS and Web of Science databases were searched in September and October 2018.

**RESULTS:**

The findings showed that RIME promoted resignification of the symbolic pain of the death of patients without the possibility of cure; improved quality of life within the process of dying; contributed to the quality of life of breast cancer patients with cure possibilities; contributed to the emotional wellbeing of ostomized patients; brought quality-of-life benefits for patients with head-and-neck cancer; promoted empowerment for women with breast cancer and strengthened their libido; and promoted resignification of the spiritual pain of bereaved youths, offering a satisfactory return from mourning preparation.

**CONCLUSIONS:**

It was found that RIME has a construct history based on rigorous scientific methodology, covering quality of life and spiritual, emotional and subjective wellbeing. RIME has not been used internationally and new studies within this field, with different cases, should be encouraged.

**SYSTEMATIC REVIEW REGISTRATION:**

PROSPERO ID 164211.

## INTRODUCTION

High levels of stress lead to problems that compromise health, quality of life and individual productivity and consequently act to trigger diseases. These problems are some of the reasons that have led to research on methods for minimizing the harmful effects of stress.^1^

Integrative and complementary practices, also known as traditional and complementary medicines, are a set of healthcare practices that encompass the traditions of care practiced in different cultures and other complementary practices that are not included in those traditions, but are incorporated into the healthcare system.^2^ These practices are characterized by procedures that have the aim of producing psychophysical and logical relaxation, and they involve creation of therapeutic bonds and integration of individuals with their environment.^2^

In a randomized clinical trial on 60 patients with chronic kidney disease in which the objective was to evaluate the therapeutic effect of music on the anxiety and vital parameters of those patients, the results showed that music therapy, which is one of the integrative and complementary practices that have recently been introduced into the Brazilian National Health System (Sistema Único de Saúde, SUS), significantly reduced their anxiety.^3^

Through integrating techniques of relaxation, directed imagination and elements that make up spirituality, a brief intervention named “relaxation, mental images and spirituality” (relaxamento, imagens mentais e espiritualidade, RIME) was developed. RIME is a form of complementary and alternative health therapy and is a pioneer in relating elements of spirituality to relaxation and to visualization of mental images.^4^ In 2005, a training program was developed for use of this intervention by other healthcare professionals, with analysis on these professionals’ experience during its application and evaluation among patients.^5^ Since the time of this training experience, the technique has gained the abbreviation RIME and has begun to be considered to be a psychotherapeutic intervention.^5^

RIME is a brief psychotherapeutic intervention for terminally ill patients, and for patients with chronic diseases that have curative possibilities, within a palliative care context. The purpose of this intervention is to improve the patients’ wellbeing, through stimulating positive transformations that come from within the individual.^6^

The resignification of spiritual pain through the RIME intervention integrates two techniques for addressing questions of spirituality: mental relaxation and visualization of mental images. The association of these two techniques favors deeper contact with individuals’ internal and personal realities, which enables them to change their attitudes, conceive new ideas and elaborate new meanings, in the light of events.

As healthcare professionals, we can promote bringing these practices together for those who can benefit from them, especially for individuals who are physically and psychologically debilitated. Such actions form part of holistic humanization behavior.

The questions that guided this study were: In what contexts has the brief psychotherapeutic intervention “relaxation, mental imagery and spirituality” been used? What were its benefits?

## OBJECTIVE

The purpose of this review was to ascertain the history, use and benefits of the RIME brief psychotherapeutic intervention that have been reported in the scientific literature in experimental or non-experimental studies within different health/disease contexts.

## METHODS

### Design and setting

The methodological design for this study consisted of a systematic review of the literature that was conducted in accordance with the Preferred Reporting Items for Systematic Reviews and Meta-Analyses (PRISMA) methodology, as proposed by Moher et al.^7^

### Search strategy

The searches were carried out in the following databases: BVSPsi, CINAHL, MEDLINE Complete, SciELO, SCOPUS and Web of Science. The descriptors were chosen and identified in accordance with the MeSH list of descriptors, as follows: *Relaxation, Mental Images* and *Spirituality.* Two combinations were performed, of which the first was: (“*Relaxation*” AND “*Mental Images*” AND “*Spirituality*”). The second combination was composed only of the intervention abbreviation: (“*RIME*”). The same search strategies were used in all databases (Table 1). This review was conducted in September and October 2018.

**Table 1.** Search strategies used in the databases BVSPsi, CINAHL, MEDLINE Complete, SciELO, SCOPUS and Web of Science

BVSPsi	1 “*Relaxation*” AND “*Mental Images*” AND “*Spirituality*” 2 “RIME”
CINAHL	1 “*Relaxation*” AND “*Mental Images*” AND “*Spirituality*” 2 “RIME”
MEDLINE Complete	1 “*Relaxation*” AND “*Mental Images*” AND “*Spirituality*” 2 “RIME”
SciELO	1 “*Relaxation*” AND “*Mental Images*” AND “*Spirituality*” 2 “RIME”
SCOPUS	1 “*Relaxation*” AND “*Mental Images*” AND “*Spirituality*” 2 “RIME”
Web of Science	1 “*Relaxation*” AND “*Mental Images*” AND “*Spirituality*” 2 “RIME”

### Eligibility selection criteria

Article identification and screening were guided by the following inclusion criteria: cross-sectional and experimental studies, which could either be randomized or not, using the RIME brief psychotherapeutic intervention method, in health/disease contexts; studies conducted on patients in any age group; patients with a probable diagnosis of any type of chronic or acute pathological condition, either in palliative care or not; studies that used the RIME intervention in association with assessment of other variables such as quality of life, resilience, wellbeing, spirituality and others; articles indexed in peer-reviewed journals; year of publication from 1999 onwards; written in the English, Spanish or Portuguese language; and full text available.

The manuscript selection was done independently and in a blinded manner by two researchers who analyzed the titles and abstracts. If these were found not to be related to the proposed theme, or not to fit the inclusion criteria, they were excluded. After this stage, a consensus meeting was held to resolve doubts and possible disagreements regarding the data collected, based on the PRISMA protocol.

## RESULTS

A total of 102 studies were selected, through the search in the databases. From the first combination, (“*Relaxation*” AND “*Mental Images*” AND “*Spirituality*”), 20 articles were identified: BVSPsi = 1; CINAHL = 2; MEDLINE = 3; SciELO = 4; SCOPUS = 6; and Web of Science = 4. From the second combination, (“*RIME*”), 82 articles came up: BVSPsi = 6; CINAHL = 68; and SciELO = 8. Four additional articles were also selected from other sources, through Google Scholar, making an overall total of 106 articles.

**Figure 1.**
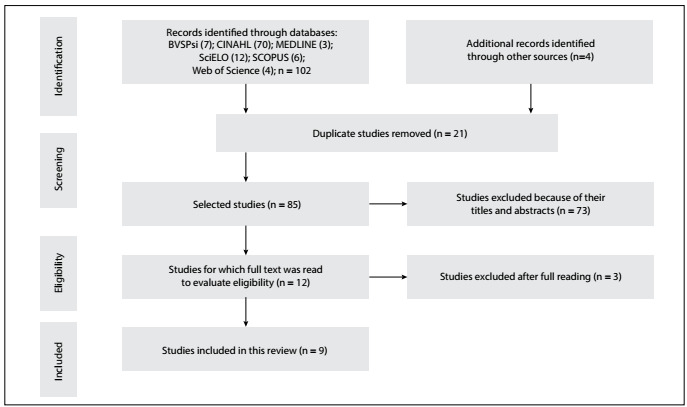
Article selection and identification process.

As shown in Figure 1, 21 repeated articles were removed, thus leaving 85 titles and abstracts to read. After the various exclusions, 12 studies were selected to make up this review. However, three more articles were subsequently excluded, after reading them fully, because they were found to consist of abstracts indexed in congress annals. Hence, in the end, this review was conducted on nine studies.

The material identified in this review is summarized in Table 2. It consisted of seven articles, one master’s degree thesis and one doctoral degree thesis. The two theses came from other sources (i.e. not the databases), as previously mentioned.^4^^,^^5^^,^^8^^,^^10^^,^^11^^,^^12^^,^^13^^,^^14^^,^^15^

**Table 2.** Summary of studies selected for this review

Author/year	Study location	Design	n	Age	Main findings
Elias et al.^4^	Campinas (SP)	Qualitative approach - clinical case study, longitudinal	5	37-75 years	Cancer patients who received two to four relaxation, mental image and spirituality sessions achieved better quality of life within the process of dying. It was observed that application of this technique was important for resignification of the symbolic pain of these patients’ deaths.
Elias^8^	Campinas (SP)	Qualitative approach	7	From 22 months to 17 years	In a qualitative study, four children and three adolescents with cancer that was beyond the possibilities of cure, each received three to ten sessions of relaxation, mental images and spirituality. After the visits, it was observed that the intervention gave new meaning to the symbolic pain of death and provided quality of life within the process of dying for these patients.
Elias et al. ^5^	Campinas (SP)	Qualitative/quantitative	6	**	Development of a training course to instruct healthcare professionals about the use of RIME intervention. Qualitative results were analyzed through content analysis, semi-structured interviews and a diary; the quantitative data were analyzed through a descriptive method, using the Wilcoxon test. The program proved to be effective in preparing healthcare professionals to using RIME intervention, enabling them to care and to provide spiritual assistance from an academic perspective.
Elias et al.^10^	Campinas (SP)	Quantitative/qualitative	11	27-76 years	A study conducted among cancer patients who received RIME intervention. Through a qualitative approach, six categories and eleven subcategories were found, among which the most prevalent were: fear of death due to denial of the severity of the clinical picture; fear of death due to perceiving the severity of the clinical picture; and fear of disintegration of feeling and of being affectively forgotten after death. Through a quantitative analysis, a statistically significant difference was observed (P < 0.0001). The results suggested that RIME promoted quality of life within the process of dying, with serenity and dignity before death.
Ribeiro et al.^11^	São Paulo (SP)	Quantitative/qualitative	21	61.3 years*	Patients using intestinal ostomies during an immediate postoperative period marked a visual analogue scale regarding wellbeing before and after RIME application: the respective mean scores were 3.33 and 1.38. Through asking the patients how they felt emotionally before the surgery and after the RIME intervention, the following context units emerged: feelings, emotions, sensations and expectations of action, which generated four distinct categories that represented the transformation relating to mental wellbeing. RIME was the only variable that presented statistical significance, which led to the affirmation that it contributed to improvement of the patients’ emotional wellbeing.
Elias et al.^12^	São Paulo (SP)	Randomized controlled study/quantitative/qualitative	28	33-59 years	The women were screened and randomized through five draw groups. In each group, half of the patients went to the control group to receive up to twelve sessions of brief psychotherapy (BP) and the other half went to the RIME group to receive three sessions of this plus twelve BP sessions. The qualitative data were treated using branched content analysis into thematic analysis; the quantitative data were collected using the WHOQOL brief scale, Rosenberg’s self-esteem scale, Beck’s hopelessness scale and a visual analogue wellbeing scale. The results showed that RIME promoted a significant improvement in quality of life perception (38.3%), compared with the control group (12.5%), along with a significant improvement in the patients’ self-esteem (14.6%).
Espinha^13^	Marília (SP)	Randomized controlled study	44	57-63 years	A study conducted among patients with head-and-neck cancer, using the performance scale instrument (ECOG) the QOL questionnaire EORTC-QLQ-C30 and the QLQ-H&N35 questionnaire for patients with head-and-neck neoplasia. The results suggested that the RIME intervention led to quality-of-life benefits for patients with head-and-neck cancer, regardless of the toxicity of the radiotherapy treatment.
Elias et al.^14^	São Paulo (SP)	Comparative exploratory, quantitative/qualitative approach	28	33-59 years	Implementation of the RIME brief psychotherapeutic intervention among women with breast cancer who were undergoing treatment, with the possibility of cure. The main focus of the study was to present the qualitative results, for which the instruments used were recorded semi-structured interviews and graphic representations from before the first and after the third RIME session. The results showed that RIME promoted empowerment for higher libido and constructive strength among women with breast cancer with the potential for cure.
Pereira^15^	Fortaleza (CE)	Qualitative research	4	16-17 years	The RIME intervention was used to resignify the pain of symbolic loss among four adolescents who were in a context of vulnerability. The analysis from the RIME brief psychotherapeutic intervention was done through observation of the spiritual pain and its intensity, as manifested by the research subjects, along with their experiences of pain resignification. For this, two instruments were used: a visual analogue scale of wellbeing and content analysis through the thematic analysis technique. The RIME intervention promoted resignification of the spiritual pain of these young mourners, thus offering a satisfactory return from mourning and providing possibilities for working to break ties and recurrence of pain.

*Average age; **unspecified age.

RIME = relaxation, mental images and spirituality (relaxamento, imagens mentais e espiritualidade); WHOQOL = World Health Organization Quality of Life; ECOG = Eastern Cooperative Oncology Group; QOL = Quality of Life; EORTC = European Organization for Research and Treatment of Cancer.

## DISCUSSION

In order to provide a detailed and uniform profile of the use of RIME, the studies will be approached sequentially, as shown in Table 2.

Elias and Giglio^4^ studied the effectiveness of the RIME intervention for terminal patients. This was developed through integrating techniques for mental relaxation and visualization of mental images with the elements that make up spirituality, with the purpose of resignifying the symbolic pain of death, as represented by mental and spiritual pain. These authors investigated five adult women aged 37 to 75 years who had been diagnosed with breast cancer at a non-curable stage. One putative variant that was studied was quality of life, and the intervention variables were “mental pain”, represented by humor, fear, “depressive” ideas and death; and ideas and conceptions regarding spirituality, the meaning of life and death and God.

These authors followed the steps of identification of the symbolic pain of death through a semi-structured interview, in which the elements of mental and spiritual pain, mental relaxation techniques and visualization of mental image orientation were condensed. It was observed that it was possible to obtain good results through applying the methods of mental relaxation, mental imagery and spirituality during the period that followed the phase of moving beyond the possibility of cure. The integration of relaxation techniques, visualization of mental images and elements that composed spirituality was important for resignifying the symbolic pain of the death of patients who were beyond the possibility of cure.

Based on reports from patients who had undergone a near-death experience that suggested an occurrence of transcendence, Elias^8^ developed the RIME intervention pilot project in 1988. The study was conducted on four children and three adolescents aged 22 months to 17 years, all with cancer and without the possibility of cure. A mental image visualization technique was developed among these children through the instruments of graphic activities, games and children’s stories; and among these adolescents, through young-adult and youth stories and films (with a plot containing a symbolic relationship with the patient’s mental and spiritual pain) and through visualization itself.

Elias^8^ observed that this proposed method, i.e. integration of the techniques of mental relaxation and mental image visualization with the elements that make up spirituality, favored resignifying the symbolic pain of death among the seven patients, since all of them would now go on to die with moral dignity, in a state of emotional support and in peace. It was concluded that the proposed method provided quality of life within the process of dying, thus enabling a serene and dignified death.

In this study (Elias^8^), in comparing follow-ups performed among adolescents and children who were within the process of dying, it was observed that the adolescents facing the symbolic pain of death presented both mental and spiritual pain, but the children presented only mental pain, represented by a depressive mood that was linked to anxiety about separation. This difference was attributed to these children’s cognitive stage.

On the basis of Piaget’s studies, Elkind^9^ stated that children from two to seven years old are in the preoperational thinking stage, whereas from seven to eleven years of age they are at the stage of concrete operational thinking. This would mean that children at all of these ages (two to eleven years) have not yet developed the ability to think abstractly. Such thinking is necessary in order to feel fear of death and the time after death, and to develop ideas and conceptions relating to spirituality.

Between 2002 and 2005, while researching for a doctoral degree thesis, Elias developed a training course to instruct healthcare professionals about the use of the RIME intervention. This was described by Elias et al.^5^ using qualitative methodology, phenomenology and action research as the theoretical bases. The training course was put into operation for use in interventions and the experience of these professionals during its application and evaluation among patients was analyzed. Six professionals participated in the study: a nurse, a doctor, three psychologists and an alternative therapist. All of them had qualifications relating to palliative care. These professionals were invited to apply the intervention to 11 cancer patients aged 27 to 76 years, who were either in public hospitals and in their homes, in the cities of Campinas, Piracicaba and São Paulo in Brazil. From this training, the technique gained the abbreviation RIME and began to be considered to be a brief psychotherapeutic intervention.

Administration of RIME revealed statistically significant differences in wellbeing levels. The patients reported having higher wellbeing levels at the end of the sessions than at the beginning (P < 0.0001), thus suggesting that RIME led to resignification of spiritual pain for these terminal patients. The proposed training program proved to be effective for preparing healthcare professionals to implement RIME interventions, both for taking care of such patients and for providing spiritual assistance from an academic perspective.^5^

Elias, Giglio & Pimenta^10^ conducted a qualitative study based on phenomenology and quantitative descriptions, in which the aim was to study the nature of spiritual pain and its resignification during application of RIME interventions. The sample consisted of 11 patients with final-stage cancer that was being treated in public hospitals, by six professionals who had been trained to apply RIME. The most prevalent categories of feelings were the following: fear of death due to the individual’s denial of the severity of his or her clinical state (n = 5); fear of death due to perceiving the severity of the clinical picture (n = 5); and fear that after death there would be disintegration of feeling, non-existence and being affectively forgotten (n = 5). These results suggested that RIME promoted quality of life during the process of dying, with serenity and dignity before death.

Ribeiro et al.^11^ conducted a qualitative study in which they aimed to evaluate and discuss the efficacy of the RIME brief psychotherapeutic intervention for wellbeing, in a group of patients who were using intestinal ostomies during a postoperative period. Twenty-one patients participated in the sample and were assessed regarding their wellbeing using a visual analogue scale before and after RIME application. Their mean scores were 3.33 before and 1.38 after the intervention. From asking these patients how they felt emotionally before the surgery and after the RIME intervention, the following context units were extracted: feelings, emotions, sensations and expectations of action. These generated four distinct categories that represented the transformation relating to mental wellbeing. It was concluded that RIME was the only variable that presented statistical significance, which led to affirmation that it contributed towards improving these ostomized patients’ emotional wellbeing.

Elias et al.^12^ conducted a study with the objective of ascertaining the benefits from RIME among 28 women who had been diagnosed with breast cancer with the possibility of cure. They had been mastectomized and were in the process of breast reconstruction with adjuvant treatments. These women were screened and randomized through five draw groups, and in each group, half of the patients went to the control group to receive up to twelve sessions of brief psychotherapy (BP) and the other half went to the RIME group to receive three sessions of this plus twelve BP sessions.

From the statistical analysis comparing the RIME group with the control group, a significant improvement (38.3%) in quality-of-life perception on the World Health Organization Quality of Life (WHOQOL) scale after RIME, compared with BP alone in the control group (12.5%), and with BP in the RIME group (16.2%). There was a significant improvement in self-esteem after RIME (14.6%), compared with BP alone in both the control group (worsened by 35.9%) and BP in the RIME group (8.3%). There were similar improvements regarding hopelessness in the RIME group and in the control group: RIME = 20.1%; RIME + BP = 27.1%; and BP = 11.1%. There was significant improvement in wellbeing relating to focused distress (measured using a visual analogue scale), both in the RIME group (poor wellbeing to wellbeing) and in the control group (very poor wellbeing to good wellbeing). None of the three treatments (RIME, RIME + BP or BP) showed any significant improvement in the WHOQOL domains or in WHOQOL health satisfaction.^12^

In another randomized controlled clinical study,^13^ conducted in Marília, state of São Paulo, the aim was to evaluate the efficacy of the RIME brief psychotherapeutic intervention among 44 patients with head-and-neck cancer, in relation to physical symptoms and quality-of-life levels. Patients in the control group only received the standard support treatment that was routinely used in that hospital unit for patients with head-and-neck cancer.

All the participants underwent performance status measurements using the Eastern Cooperative Oncology Group (ECOG) scale. They answered a questionnaire regarding their profile and, at both the start and the end of the study period, answered questions relating to the quality-of-life evaluation of the European Organization for Research and Treatment of Cancer Quality-of-Life Questionnaire C30 (EORTC-QLQ-C30) and the EORTC-QLQ-H&N35 questionnaire. The QLQ-H&N35 (Quality-of-Life Questionnaire “Head-and-Neck Module” 35 items) questionnaire is a specific module of the EORTC-QLQ-C30 that is intended for patients with head-and-neck neoplasms, at various stages and undergoing different treatments.

In addition to support treatment, the experimental group underwent the RIME therapeutic intervention periodically. In the intervention group, subjects who underwent RIME showed significant improvements in most domains of the general and specific quality-of-life scales at the end of the radiotherapy treatment. Furthermore, there was lower consumption of analgesic drugs and less weight reduction at the end of the treatment. The results suggested that the RIME brief psychotherapeutic intervention provided quality-of-life benefits for patients with head-and-neck cancer, regardless of the toxicity that resulted from the radiotherapy treatment.

Elias et al.^14^ conducted a randomized study on implementation of RIME for women undergoing breast cancer treatment, with the possibility of cure. The main focus of this study was to present qualitative results from recorded semi-structured interviews and graphic representations that were conducted before the first and after the third RIME session.

Qualitative analysis on these data indicated that the main issue among the women with breast cancer in the RIME group, i.e. the focus for transformation, was the need for self-valorization. This was also observed in the BP control group. The main psychological transformations mediated by the RIME symbolic elements were the following:

1. transformation of female absence or dissipation to loving or protective representation;
2. transformation of masculine intangibles, absence or impotence to tangible, powerful and loving representation; and
3. transformation of divine intangibles that are inaccessible or impersonal to close-at-hand, accessible and loving representation.

In a qualitative study, conducted in Fortaleza, state of Ceará, Pereira^15^ used RIME interventions in order to resignify losses caused by deaths. In this study, a psychopedagogical device that would resignify the spiritual pain of loss was proposed, in order to help four young mourners deal with the deaths of people for whom they had affectionate ties who had been lost through acts of violence. The four individuals studied were school-age adolescents aged between 16 and 17 years who were living in a region of extremely high vulnerability, where deaths occur very frequently, mainly through violence.

This author took mourning to be a reactive psychological process that constituted an adaptation to rupturing of bonds, and considered that the bereaved individual would be an active subject in the processes of facing up to ruptures and losses. Thus, this author chose life history and training and the RIME intervention as the research method. Through integrating these two forms of access, with formative and transformational self-knowledge, the results showed that even if the rupturing of a bond through death provokes inevitable pain, it can be resignified to acquire a providing sense: new ways of living, loving and dealing with finitude.

Each of these adolescents received three sessions of the intervention, which was applied once a week. RIME was used with the aim of resignifying the symbolic pain of the loss suffered by these four adolescents, in their context of vulnerability. The analysis on the results from the RIME brief psychotherapeutic intervention was made through observation of the spiritual pain and its intensity that the study subjects manifested, along with their experiences of resignification of this pain. For this, two instruments were used: a visual analogue scale of wellbeing and content analysis through a thematic analysis technique.

In specific relation to the RIME intervention, this promoted resignification of the spiritual pain of the bereaved young people, thus offering a satisfactory return from mourning and providing possibilities for working on the rupturing of ties and the recurrent pain from these ties.^15^

## CONCLUSIONS

It was found that RIME has a construct history based on rigorous scientific methodology, and that it has become consolidated from the time of its creation to the present day, including n relation to quality of life and emotional and subjective wellbeing, in different health-disease contexts.

The pathological conditions that have been studied using this method have included patients with breast cancer who were beyond the curable stage; patients with other types of cancers, both beyond and within the possibilities for cure; patients using intestinal ostomies during an immediate postoperative period; and patients with head-and-neck cancer. This intervention has also been applied to bereaved adolescents within a context of vulnerability, who needed to elaborate resignification of spiritual pain and to work on the rupturing of bonds and the pain arising from this.

The RIME brief psychotherapeutic intervention promoted resignification of the symbolic pain of death among patients who were beyond the possibility of cure; improved quality of life within the process of dying; and contributed towards improve emotional wellbeing among ostomy patients. It provided quality-of-life benefits for patients with head-and-neck cancer, regardless of the toxicity of radiotherapy treatment. It promoted empowerment for strengthening libido and constructive force among women with breast cancer for whom cure was possible, and promoted resignification of the bereavement of young men in spiritual pain.

The RIME intervention provides a major contribution to science and the humanization of healthcare. One limitation identified in the present study was that we found that this intervention has not yet been used internationally. New studies within this field, covering different cases, should therefore be encouraged.
